# Predicted the *P2RX7* rs3751143 polymorphism is associated with cancer risk: a meta-analysis and systematic review

**DOI:** 10.1042/BSR20193877

**Published:** 2021-02-03

**Authors:** Bi-jun Wang, Jun-yi Chen, Yu Guan, Da-chao Liu, Zi-chuan Cao, Jin Kong, Zheng-Sheng Wu, Wen-Yong Wu

**Affiliations:** 1 Department of General Surgery, The First Affiliated Hospital of Anhui Medical University, Hefei, Anhui, China; 2 Department of Urology, The First Affiliated Hospital of Anhui Medical University, Institute of Urology, Anhui Province Key Laboratory of Genitourinary Diseases, Anhui Medical University, Hefei, PR China; 3 Department of Pathology, Anhui Medical University, Hefei, Anhui, China

**Keywords:** cancer, meta analysis, P2RX7, polymorphism, rs3751143

## Abstract

**Background:** Both meta-analyses and systematic reviews were used to assess the relationship between purinergic receptor P2X ligand-gated ion channel 7 (*P2RX7*) rs3751143 polymorphism and the risk of cancer.

**Materials and methods:** The data used in this research were collected from Google Scholar, Web of Science, CNKI, and Wan Fang Data databases. The final retrieval ended on 22 February 2019. The strength of correlation was assessed using odds ratios and 95% confidence intervals. Based on the heterogeneity test results, fixed-effect (Mantel–Haenszel) or random-effects (DerSimonian–Laird) models were selected to summarise the collective effects.

**Results:** Eight separate studies containing 1462 cancer cases and 3037 controls were enrolled. Overall, there was no significant association between *P2RX7* rs3751143 polymorphism and the risk of cancer in the allelic, homozygous, heterozygous, dominant, or recessive models.

**Conclusions:** Our meta-analysis indicates that there is no significant association between *P2RX7* rs3751143 polymorphism and the risk of cancer in the allelic, homozygous, heterozygous, dominant, and recessive models.

## Introduction

Cancer is a global public health issue, and a significant proportion of deaths each year are caused by cancer. According to the studies, one in four people in the United States died of cancer [1]. In recent years, many theories have been proposed for the pathogenesis of cancer, including gene mutation [2,3], oxidative stress [4,5], and ionizing radiation theory [6], owing to extensive long-term research worldwide. In addition, several single-nucleotide polymorphisms (SNPs) in different genes have been found to be specific biomarkers in various cancers; for example, Sebastian et al*.* [7] demonstrated that SNP is a risk factor for cervical carcinogenesis in Caucasian Polish individuals.

The *P2X7* gene, also known as the *P2RX7* gene, is located on chromosome 12, a highly polymorphic gene with more than 11 polymorphisms that have specific impacts on *P2RX7* function, including loss of function or feature enhancement [8]. Furthermore, *P2RX7* polymorphisms are clinically critical in various diseases, including tumors [9,10]. Some studies have indicated the clinical significance of *P2RX7* polymorphism in tuberculosis and mood disorders [11–16]. Wesselius et al*.* [7] believe that two genetic polymorphisms of the *P2RX7* gene (Glu496Ala and Gly150Arg) are linked to an increased risk of osteoporosis. Gadeock et al*.* [17] demonstrated an association between *P2RX7* genetic polymorphisms and organic cation uptake in human myeloid leukaemia kg-1 cells, and they found that these functional polymorphisms can result in the verification of the survival and invasiveness of myeloid leukaemia cells.

Thus far, most studies have only focussed on the non-synonymous polymorphism rs3751143 (A>G, Supplementary Figure S1), which was identified as the highest associated polymorphism in two published studies [11,12]. The 1513 (1513A>C) A to C polymorphism, which leads to the replacement of Glu-496 at the C-terminal tail of the cell with an Ala residue and causes the loss of function [18] of the receptor, is of particular interest. This polymorphism is linked to familial papillary thyroid carcinoma (PTC) [19], hepatocellular carcinoma (HCC) [9], and chronic lymphocytic leukaemia (CLL) [20–22]. Nevertheless, the data from these published case–control studies were not consistent. One study may be insufficient to identify slight influences of these polymorphisms on cancer susceptibility. Therefore, we conducted a comprehensive meta-analysis and systematic review to explore the relationship between *P2RX7* rs3751143 polymorphism and the risk of cancer.

## Methods

### Search strategy and study selection

The data in this research were obtained from Google Scholar, Web of Science, CNKI, and Wanfang databases. The final retrieval ended on 22 February 2019 using the following retrieval types: ((*P2RX7* OR *P2X7*) AND (polymorphism OR mutation OR variation OR single nucleotide polymorphism OR genotype) AND (cancer OR tumour OR tumour OR malignant tumour OR neoplasm).

Articles that satisfied the following inclusion criteria were included in the current study: (1) studying the relationship between *P2RX7* polymorphism and the risk of cancer, (2) case–control design, (3) extracting genotype and allele frequencies to calculate advantage odds ratio (OR) and 95% confidence interval (CI), and (4) full-text articles published in English or Chinese. The major exclusion criteria were as follows: (1) case report, case-solo, or retrospective studies and (2) studies without primary data on *P2RX7* polymorphisms (or contact with the corresponding author still did not achieve the necessary raw data).

### Data extraction

Two reviewers extracted data independently using standard protocols, and the results were reviewed by a third reviewer (Bijun Wang, Junyi Chen, and Yu Guan). These distinctions were resolved through a discussion with the research team. We extracted data on the first author, publication year, journal, ethnicity, type of cancer, number of cases, control groups, and available genotypes and allele frequencies for the *P2RX7* polymorphisms from each study. If no there was no raw data in the related articles, an additional data request was sent to the corresponding author.

### Statistical analyses

Statistical analyses were performed using STATA version 12.0 (StataCorp, College Station, TX, U.S.A.). ORs and 95% CIs were used to evaluate cancer susceptibility. In addition, pooled ORs of the following five genetic models were computed: allelic contrast, heterozygote, homozygote, dominant, and recessive models. The heterogeneity of various studies was measured using a *Q*-test based on the chi-square test. The Hardy–Weinberg Equilibrium (HWE) of the control group was calculated using the chi-square test. Sensitivity analysis was used to evaluate the stability of the results, and the publication bias was evaluated using Begg’s funnel plot and Egger’s test. Statistical significance was set at *P*<0.05. The Preferred Reporting Items for Systematic Reviews and Meta-Analyses (PRISMA) method of the present study is shown in Supplementary Table S1 [23].

## Results

### Search results

**Figure 1:**
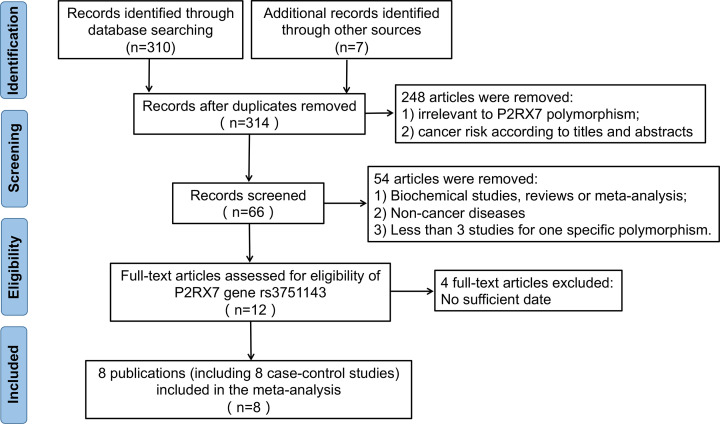
The flow diagram of included/excluded studies

As shown in Figure 1, 314 articles were retrieved after the initial research. A total of 248 articles were excluded from further screening because they were irrelevant to the study topic according to a review of titles and abstracts. Among the 66 full-text articles, reviews, or meta-analysis reviewed, 33 articles were biochemical studies; 16 articles studied non-cancer diseases such as depression; five articles analysed several *P2RX7* polymorphisms that were not studied in other independent studies, resulting in the impossibility of data pooling; and four articles failed to provide sufficient data for calculation. Therefore, we eventually enrolled eight articles in this meta-analysis [9,19,20,24–29] (Table 1).

**Table 1:** Characteristics of the enrolled studies

First author	Year	Ethnicity	Genotyping method	Source of control	Cancer type	Case			Control			
						WW	MW	MM	WW	MW	MM	HWE
Dardano, A. et al*.*	2009	Caucasian	TaqMan	H-B	Papillary thyroid cancer	65	48	8	62	36	2	Y
Wiley, J.S. et al*.*	2002	Caucasian	PCR	H-B	Chronic lymphocytic leukemia	20	15	1	40	5	1	Y
Duan, S. et al*.*	2016	Asian	PCR-RPLF	H-B	Hepatocellular carcinoma	192	106	25	217	90	16	Y
Tiribelli, M. et al*.*	2004	Mix	PCR	H-B	Chronic lymphocytic leukemia	53	19	2	296	101	14	Y
Yang, Y.C. et al*.*	2016	Asian	TaqMan	H-B	Cervical squamous cell carcinoma	304	172	31	944	578	97	Y
Zhang, L.Y. et al*.*	2003	Asian	ARMS-PCR	H-B	Chronic lymphocytic leukemia	98	42	4	232	105	11	Y
Starczynski et al*.*	2002	Caucasian	PCR	H-B	Chronic lymphocytic leukemia	83	35	3	59	31	5	Y
PANEESHA, et al*.*	2005	Caucasian	PCR	H-B	Chronic lymphocytic leukemia	85	50	1	59	31	5	Y

Abbreviations: ARMS-PCR, amplification refractory mutation system-polymerase chain reaction; H-B, hospital-based; HWE, Hardy–Weinberg equilibrium; M, Minor allele type; PCR, polymerase chain reaction; PCR-RPLF, polymerase chain reaction-restriction fragment length polymorphism; W, wild t-pe.

### Characteristics of the included studies

The eight enrolled articles consisted of eight case–control studies with *P2RX7* gene rs3751143 polymorphism and four cancer types (CLL in five studies, PTC in one study, HCC in one study, and cervical squamous cell carcinoma in all studies). The ethnicities included Caucasian (four studies), Asian (three studies), and mixed (one study conducted in Australia). The control sources were all hospital-based (H-B) sources. According to the Newcastle–Ottawa Scale (NOS), five articles were of high quality (NOS score: 7–9) and three articles were of moderate quality (NOS score: 5–6) (Supplementary Table S2). All studies reported the number of corresponding genotypes for both the cases and control groups as recessive, heterogeneous, and wild genotypes.

### Quantitative analysis

The pooled ORs and corresponding 95% CIs are shown in Table 2. Eight studies, including 1462 cases and 3037 controls, were evaluated for *P2RX7* rs3751143 polymorphism. Overall, analyses showed that no evident risk of cancer in the allelic, homozygous, heterozygous, recessive, and dominant models.

**Table 2:** Details of the results for current meta-analysis

Comparison	Subgroup	*P*_H_	*P*_Z_	Random	Fixed
M vs. W	Overall	0.018	0.439	1.086 (0.882–1.337)	1.051 (0.939–1.177)
	Asian	0.072	0.575	1.071 (0.843–1.361)	1.042 (0.915–1.188)
	Caucasian	0.009	0.463	1.220 (0.717–2.074)	1.102 (0.855–1.421)
	PCR	0.025	0.754	1.081 (0.664–1.761)	0.975 (0.752–1.265)
	TaqMan	0.097	0.592	1.110 (0.757–1.628)	1.007 (0.862–1.177)
	CLL	0.052	0.941	1.013 (0.719–1.427)	0.964 (0.780–1.190)
MM vs. WW	Overall	0.179	0.621	1.062 (0.664–1.699)	1.079 (0.798–1.459)
	Asian	0.311	0.42	1.162 (0.786–1.719)	1.148 (0.821–1.604)
	Caucasian	0.063	0.793	0.818 (0.183–3.666)	0.850 (0.390–1.850)
	PCR	0.442	0.106	0.514(0.209–1.1262)	0.483 (0.200–1.167)
	TaqMan	0.107	0.602	1.554 (0.446–5.406)	1.112 (0.746–1.658)
	CLL	0.531	0.14	0.622 (0.305–1.269)	0.589 (0.292–1.190)
MW vs. WW	Overall	0.058	0.355	1.114 (0.886–1.401)	1.056 (0.917–1.217)
	Asian	0.196	0.89	1.035 (0.821–1.305)	1.012 (0.857–1.195)
	Caucasian	0.023	0.288	1.375 (0.764–2.475)	1.226 (0.900–1.670)
	PCR	0.023	0.378	1.306 (0.721–2.365)	1.157 (0.848–1.580)
	TaqMan	0.292	0.710	0.974 (0.772-1.230)	0.963 (0.789–1.175)
	CLL	0.041	0.483	1.164 (0.761–1.780)	1.080 (0.840–1.388)
MW + MM vs. WW	Overall	0.027	0.365	1.116 (0.880–1.414)	1.060 (0.926–1.213)
	Asian	0.104	0.704	1.062 (0.816–1.384)	1.031 (0.880–1.208)
	Caucasian	0.013	0.344	1.339 (0.732–2.447)	1.181 (0.876–1.592)
	PCR	0.027	0.365	1.116 (0.880–1.414)	1.060 (0.926–1.213)
	TaqMan	0.164	0.858	1.058 (0.732–1.531)	0.983 (0.813–1.188)
	CLL	0.036	0.647	1.102 (0.727–1.671)	1.023 (0.803–1.305)
MM vs. MW + WW	Overall	0.261	0.67	1.054 (0.695–1.600)	1.067 (0.792–1.438)
	Asian	0.46	0.43	1.140 (0.815–1.595)	1.142 (0.821–1.590)
	Caucasian	0.084	0.693	0.752 (0.182–3.107)	0.808 (0.372–1.751)
	PCR	0.083	0.092	0.499 (0.204–1.218)	0.469 (0.195–1.130)
	TaqMan	0.140	0.537	1.476 (0.491–4.439)	1.132 (0.764–1.678)
	CLL	0.588	0.129	0.615 (0.303–1.248)	0.582 (0.289–1.171)

Abbreviations: CLL, chronic lymphocytic leukemia; H-B, hospital-based; HWE, Hardy–Weinberg equilibrium (Y: conformed to HWE); M, minor allele type; *P*_H_, *P*-value for heterogeneity test; *P*_Z_, *P*-value for significance test; W, wild-type.

With regard to the stratification analysis by ethnicity, genotyping method, and HWE status, the results were consistent with the overall result that there was no correlation between *P2RX7* rs3751143 polymorphism and the risk of cancer.

### Sensitivity analysis and publication bias

**Figure 2:**
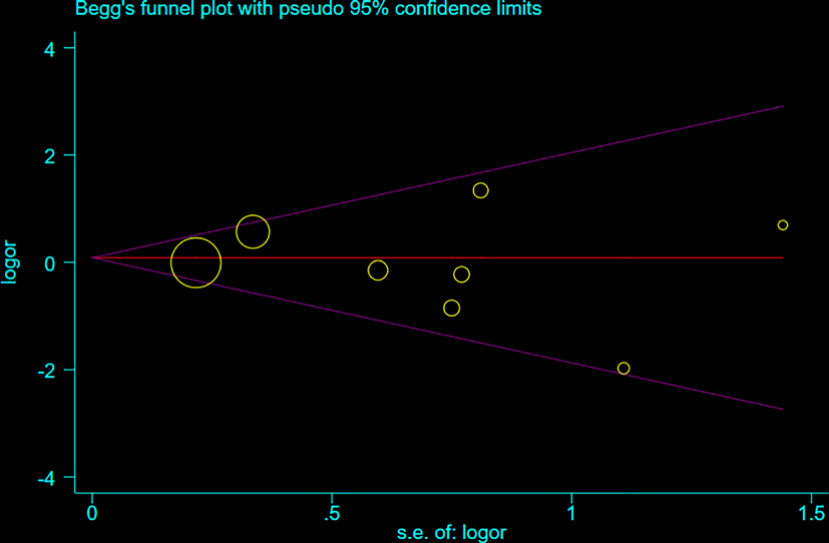
Begg’s funnel plot for *P2RX7* gene rs3751143 polymorphism (M vs. W) The *x*-axis log (OR) and the *y*-axis is natural logarithm of OR. The horizontal line in the figure represents the overall estimated log (OR). The two diagonal lines indicate the pseudo 95% confidence limits of the effect estimate. Log (OR) = log-transformed OR, OR = odds ratio.

This meta-analysis was repeated and we omitted each study individually to test the effectiveness of all eligible studies included in our study. The results indicated no substantial change in the set ORs corresponding to rs3751143 polymorphism (Supplementary Table S3). Publication bias was assessed using funnel plots generated by STATA 12.0. Any obvious asymmetry was hardly observed (Figure 2). We further conducted Egger’s regression test for the overall and subgroup analyses, and the results demonstrated no significant publication bias (Supplementary Table S4, *P*>0.05).

## Discussion

*P2RX7* has been investigated for its role in conferring susceptibility to various emotional disorders. It is located in 12q24 and contains 13 exons spanning the 53-kb region [30]. *P2RX7* encodes a purinergic receptor involved in the Ca^2+^-dependent signalling pathway. Its expression in the brain regulates immune function and neurotransmitter release [31,32]. Activation of the receptor provides inflammatory stimuli that modulate the release of proinflammatory cytokines, which have been shown to influence tumour pathogenesis in some cases. Elena et al. [33] found that *P2RX7* has a significant tumour growth-promoting effect *in vivo*. Fu et al*.* [34] also showed that *P2RX7*-mediated apoptosis activation inhibits the development of DMBA/TPA-induced mouse papilloma and cancer. However, *P2RX1, P2RX7, P2RY12, P2RY13*, and *P2RY14* were relatively downregulated in lung cancer tissues and were associated with a favourable prognosis [35].

Due to their high density, scalability, and genome-wide distribution, SNPs are considered ideal genomic resources in genetic studies for the characterisation of genetic resources and functional gene identification for traits [36]. Considerable effects on protein function and gene expression can be caused by SNPs occurring in coding regions and regulatory sequences, respectively. Therefore, SNPs have great potential in genetics, breeding, ecological, and evolutionary studies [37]. In addition to its protein function, polymorphisms may also influence tumorigenesis.

SNPs in the *P2X7R* gene have been described to influence the function of this receptor, including loss or gain of function [38–41]. Song et al. summarised the SNP arrays with regard to hematopoietic malignancies in general [42]. For instance, three *P2X7R* SNPs with amino acid changes, rs3751143 (1513A>C, Glu496Ala), rs2230911 (1096 C>G, Thr357Ser), and rs7958311 (835G>A, Arg270His), have been shown to result in a loss of receptor function in both channel and pore function [39,40]. In particular, rs3751143 polymorphism has been reported to occur in some CLL cases, resulting in loss of *P2RX7* receptor function [20]. Conversely, Starczynski *et al* [25]. analysed the *P2RX7* genotypes of 121 patients with CLL and correlated these findings with a broad range of clinical parameters, and their results indicated that *P2RX7* polymorphism of 1513 A to C is unlikely to play a vital role in the pathogenesis or progression of CLL. Hence, the incidence of this variation and its relevance to the pathogenesis of CLL is unclear. However, Thunberg et al*.* [35] found that the frequency of rs3751143 polymorphism in the normal control group was distinctly higher than that in patients with B-CLL, while the frequency of the 1513C allele was not biased between CLL patients with or without mutated VH. Compared with patients with homozygous genotype 1513A, CLL patients with heterozygous A/C polymorphisms has a significantly longer overall survival. In addition, studies have shown that *P2RX7* polymorphisms may affect sputum metabolism and play a critical role in the development of liver cancer. Duan et al*.* [9] conducted a H-B case–control study including 323 patients with liver cancer and 323 controls to investigate the relationship between *P2RX7* rs3751143 polymorphism and susceptibility to liver cancer in the Chinese Han population. Their results indicated that *P2RX7* rs3751143 polymorphism is associated with an increased risk of developing HCC.

In recent years, research related to cancer has focused on many candidate genes. However, the results of the surveys are inconsistent. To obtain sufficient detection capabilities, a large number of sample subjects is required. The lack of statistical ability in small-sample association studies may lead to contradictory results. Meta-analysis of mental illness has become critical due to the rapid rise in the number and size of datasets. In the present study, no significant association between *P2RX7* rs3751143 polymorphism and the risk of cancer in any of the genetic models was observed.

In the current meta-analysis, several specific details are worth considering. First, this meta-analysis only included published studies, which may have publication bias. Second, the results of the meta-analysis may be distorted by the discovery of significant heterogeneity between studies in some comparisons. Third, our results are based on unadjusted estimates and, if a more accurate analysis by age and sex are available in individual data. Finally, all eligible studies included Caucasian populations.

In summary, our meta-analysis showed no significant association between *P2RX7* rs3751143 polymorphism and the risk of cancer. Given the inconsistencies in the subgroup analysis of different types of cases, further studies including larger sample sizes are needed.

## Supplementary Material

Supplementary Figure S1 and Tables S1-S4

## Data Availability

The datasets used and/or analysed in this research were collected from Google Scholar, Web of Science, CNKI, and Wan fang Data databases. All data or code used during the study are available in a repository or acquired from corresponding author (https://scholar.google.com/; https://webofknowledge.com/; https://cnki.net/; http://www.wanfangdata.com.cn/index.html)

## Abbreviations

CI: confidence Interval
CLL: chronic lymphocytic leukemia
HCC: hepatocellular carcinoma
HWE: Hardy–Weinberg equilibrium
NOS: Newcastle–Ottawa Scale
OR: odds ratio
*P2RX7*: purinergic receptor P2X, ligand-gatedion channel, 7
PRISMA: Preferred Reporting Items for Systematic Reviews and Meta-Analyses
PTC: papillary thyroid carcinoma
SNP: single-nucleotide polymorphism
