# Supplementary material for: Predicted the P2RX7 rs3751143 polymorphism is associated with cancer risk: a meta-analysis and systematic review
Source: Biosci Rep. 2021 Feb 3;41(2):BSR20193877. doi: 10.1042/BSR20193877 (PMC7859319; doi:10.1042/BSR20193877)
Supplement: Supplementary Figure S1 and Tables S1-S4 [file BSR-2019-3877_supp.pdf]

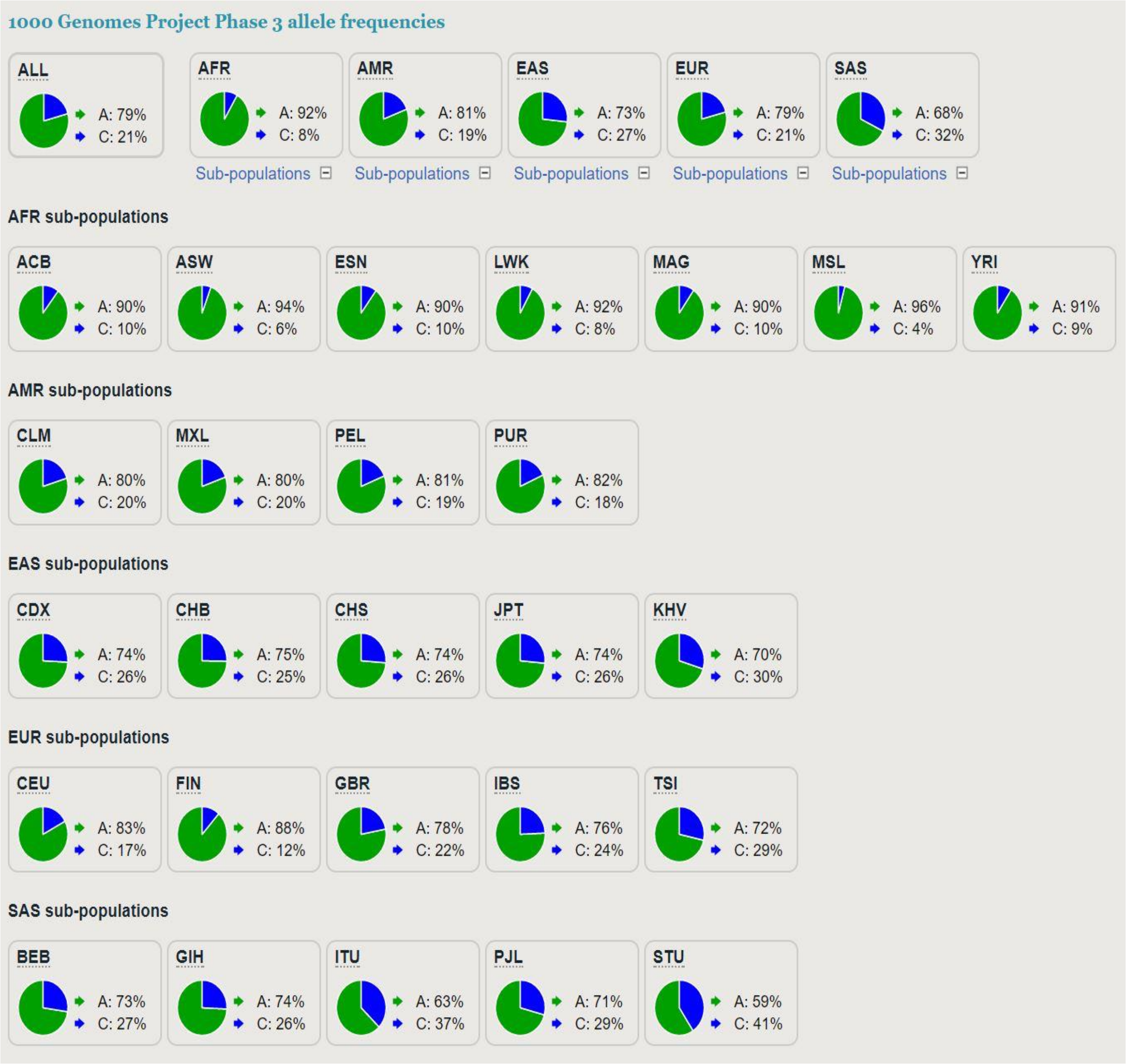
1000 Genomes Project Phase 3 allele frequencies
ALL A: 79% C: 21%
AFR A: 92% C: 8%
AMR A: 81% C: 19%
EAS A: 73% C: 27%
EUR A: 79% C: 21%
SAS A: 68% C: 32%
Sub-populations
Sub-populations
Sub-populations
Sub-populations
Sub-populations
AFR sub-populations
ACB A: 90% C: 10%
ASW A: 94% C: 6%
ESN A: 90% C: 10%
LWK A: 92% C: 8%
MAG A: 90% C: 10%
MSL A: 96% C: 4%
YRI A: 91% C: 9%
AMR sub-populations
CLM A: 80% C: 20%
MXL A: 80% C: 20%
PEL A: 81% C: 19%
PUR A: 82% C: 18%
EAS sub-populations
CDX A: 74% C: 26%
CHB A: 75% C: 25%
CHS A: 74% C: 26%
JPT A: 74% C: 26%
KHV A: 70% C: 30%
EUR sub-populations
CEU A: 83% C: 17%
FIN A: 88% C: 12%
GBR A: 78% C: 22%
IBS A: 76% C: 24%
TSI A: 72% C: 29%
SAS sub-populations
BEB A: 73% C: 27%
GIH A: 74% C: 26%
ITU A: 63% C: 37%
PJL A: 71% C: 29%
STU A: 59% C: 41%

Figure S1. *P2RX7*-rs3751143 polymorphism allele frequency from 1000 genome projects phase 3.

Table S1. PRISMA 2009 Checklist

Section/topic	#	Checklist item	Reported on page #
**TITLE**			
Title	1	Identify the report as a systematic review, meta-analysis, or both.	1
**ABSTRACT**			
Structured summary	2	Provide a structured summary including, as applicable: background; objectives; data sources; study eligibility criteria, participants, and interventions; study appraisal and synthesis methods; results; limitations; conclusions and implications of key findings; systematic review registration number.	1-2
**INTRODUCTION**			
Rationale	3	Describe the rationale for the review in the context of what is already known.	2-3
Objectives	4	Provide an explicit statement of questions being addressed with reference to participants, interventions, comparisons, outcomes, and study design (PICOS).	3
**METHODS**			
Protocol and registration	5	Indicate if a review protocol exists, if and where it can be accessed (e.g., Web address), and, if available, provide registration information including registration number.	3
Eligibility criteria	6	Specify study characteristics (e.g., PICOS, length of follow-up) and report characteristics (e.g., years considered, language, publication status) used as criteria for eligibility, giving rationale.	4
Information sources	7	Describe all information sources (e.g., databases with dates of coverage, contact with study authors to identify additional studies) in the search and date last searched.	4
Search	8	Present full electronic search strategy for at least one database, including any limits used, such that it could be repeated.	3

Study selection	9	State the process for selecting studies (i.e., screening, eligibility, included in systematic review, and, if applicable, included in the meta-analysis).	3
Data collection process	10	Describe method of data extraction from reports (e.g., piloted forms, independently, in duplicate) and any processes for obtaining and confirming data from investigators.	4
Data items	11	List and define all variables for which data were sought (e.g., PICOS, funding sources) and any assumptions and simplifications made.	4
Risk of bias in individual studies	12	Describe methods used for assessing risk of bias of individual studies (including specification of whether this was done at the study or outcome level), and how this information is to be used in any data synthesis.	4
Summary measures	13	State the principal summary measures (e.g., risk ratio, difference in means).	4
Synthesis of results	14	Describe the methods of handling data and combining results of studies, if done, including measures of consistency (e.g., I^2) for each meta-analysis.	4

Section/topic	#	Checklist item	Reported on page #
Risk of bias across studies	15	Specify any assessment of risk of bias that may affect the cumulative evidence (e.g., publication bias, selective reporting within studies).	4
Additional analyses	16	Describe methods of additional analyses (e.g., sensitivity or subgroup analyses, meta-regression), if done, indicating which were pre-specified.	4
**RESULTS**			
Study selection	17	Give numbers of studies screened, assessed for eligibility, and included in the review, with reasons for exclusions at each stage, ideally with a flow diagram.	4-5
Study characteristics	18	For each study, present characteristics for which data were extracted (e.g., study size, PICOS, follow-up period) and provide the citations.	5
Risk of bias within studies	19	Present data on risk of bias of each study and, if available, any outcome level assessment (see item 12).	7
Results of individual studies	20	For all outcomes considered (benefits or harms), present, for each study: (a) simple summary data for each intervention group (b) effect estimates and confidence intervals, ideally with a forest plot.	5-6

Synthesis of results	21	Present results of each meta-analysis done, including confidence intervals and measures of consistency.	5-6
Risk of bias across studies	22	Present results of any assessment of risk of bias across studies (see Item 15).	7
Additional analysis	23	Give results of additional analyses, if done (e.g., sensitivity or subgroup analyses, meta-regression [see Item 16]).	6-7
**DISCUSSION**			
Summary of evidence	24	Summarize the main findings including the strength of evidence for each main outcome; consider their relevance to key groups (e.g., healthcare providers, users, and policy makers).	7-8
Limitations	25	Discuss limitations at study and outcome level (e.g., risk of bias), and at review-level (e.g., incomplete retrieval of identified research, reporting bias).	7-8
Conclusions	26	Provide a general interpretation of the results in the context of other evidence, and implications for future research.	9
**FUNDING**			
Funding	27	Describe sources of funding for the systematic review and other support (e.g., supply of data); role of funders for the systematic review.	10

From: Moher D, Liberati A, Tetzlaff J, Altman DG, The PRISMA Group (2009). Preferred Reporting Items for Systematic Reviews and Meta-Analyses: The PRISMA Statement. PLoS Med 6(6): e1000097. doi:10.1371/journal.pmed1000097

For more information, visit: **www.prisma-statement.org**.

Table S2. Quality control for each enrolled publication.

Author	Year	Ethnicity	Adequacy of Case Definition	Representativeness of the Cases	Selection of Controls	Definition of Controls	Comparability Cases/Controls	Ascertainment of Exposure	Same Method of Ascertainment	Non-response rate
Starczynski et al.	2003	Caucasian	*	*	*	NA	*	*	NA	*
Thunberg et al.	2002	Caucasian	*	*	*	*	*	*	NA	*
Wiley et al.	2002	Caucasian	*	*	*	*	**	*	*	*
Zhang et al.	2003	Asian	NA	*	*	*	NA	*	NA	*
Paneesha et al.	2005	Caucasian	*	*	*	*	*	*	NA	*
Dardano et al.	2009	Caucasian	*	*	*	NA	NA	*	*	*
Duan et al.	2016	Asian	*	*	*	*	**	*	*	*
Yang et al.	2016	Asian	*	*	*	*	*	*	*	*

NA. Not Available.

Table S3. Sensitivity analysis for current work.

Comparison	Study Omitted	Estimate	95%CI	Effect Model
M vs. W	Dardano A *et al.*	1.047	0.839-1.306	Random
	Wiley J S *et al.*	1.035	0.876-1.222	
	Duan S *et al.*	1.031	0.827-1.285	
	Tiribelli M *et al.*	1.102	0.873-1.391	
	Yang YC *et al.*	1.128	0.867-1.467	
	Zhang LY *et al.*	1.117	0.877-1.421	
	Starczynski *et al.*	1.135	0.912-1.412	
	PANEESHA *et al.*	1.121	0.891-1.411	
MM vs. WW	Dardano A *et al.*	1.013	0.742-1.383	Fixed
	Wiley J S *et al.*	1.072	0.791-1.452	
	Duan S *et al.*	0.939	0.664-1.327	
	Tiribelli M *et al.*	1.094	0.804-1.489	
	Yang YC *et al.*	1.178	0.765-1.814	
	Zhang LY *et al.*	1.097	0.803-1.500	
	Starczynski *et al.*	1.127	0.828-1.534	
	PANEESHA *et al.*	1.150	0.847-1.563	
MW vs. WW	Dardano A *et al.*	1.042	0.900-1.207	Random
	Wiley J S *et al.*	1.023	0.886-1.180	
	Duan S *et al.*	1.006	0.861-1.176	
	Tiribelli M *et al.*	1.056	0.913-1.223	
	Yang YC *et al.*	1.175	0.972-1.420	
	Zhang LY *et al.*	1.070	0.921-1.244	
	Starczynski *et al.*	1.074	0.928-1.243	

	PANEESHA *et al.*	1.052	0.909-1.218	
MW+MM vs. WW	Dardano A *et al.*	1.087	0.840-1.407	Random
	Wiley J S *et al.*	1.039	0.886-1.218	
	Duan S *et al.*	1.063	0.821-1.377	
	Tiribelli M *et al.*	1.136	0.869-1.484	
	Yang YC *et al.*	1.178	0.881-1.576	
	Zhang LY *et al.*	1.158	0.878-1.526	
	Starczynski *et al.*	1.170	0.908-1.508	
	PANEESHA *et al.*	1.141	0.873-1.491	
MM vs. MW+WW	Dardano A *et al.*	1.006	0.740-1.369	Fixed
	Wiley J S *et al.*	1.065	0.789-1.437	
	Duan S *et al.*	0.949	0.674-1.336	
	Tiribelli M *et al.*	1.082	0.797-1.468	
	Yang YC *et al.*	1.117	0.727-1.714	
	Zhang LY *et al.*	1.083	0.795-1.475	
	Starczynski *et al.*	1.110	0.818-1.506	
	PANEESHA *et al.*	1.138	0.840-1.541	

95%CI: 95% confidence Interval.

Table S4. Publication bias for current work.

**Subgroup analyses**	**Egger's test P > |t|**
Overall	0.663
Caucasian	0.918
Asian	0.899
CLL	/
Y	/

CLL: Chronic Lymphocytic Leukemia; **Y:** conformed to HWE.
